# ‘Testicular masquerade’: a case report of testicular malignancy with persistent Müllerian duct syndrome and transverse testicular ectopia

**DOI:** 10.1308/rcsann.2023.0088

**Published:** 2023-12-01

**Authors:** D Barki, N Manayath, BS Vatsa, S Venkatanarasimhan N, V Vishnuvardhana G, S Achar, B Bhat

**Affiliations:** Bangalore Baptist Hospital, India

**Keywords:** Persistent Müllerian duct syndrome (PMDS), Transverse testicular ectopia (TTE), Testicular malignancy, Mixed germ cell tumour, Undescended testis

## Abstract

Persistent Müllerian duct syndrome (PMDS) is a rare sexual development disorder. It is even more rarely associated with transverse testicular ectopia (TTE), a rare form of testicular ectopia, in which both testes descend through a single inguinal canal and are present in the same hemiscrotum. PMDS with TTE is associated with 18%–33% malignant transformation. Here we report the case of a 48-year-old man who presented with a large right inguinoscrotal swelling and on evaluation was found to have a large right testicular mass with complete right inguinal hernia, undescended left testis and a central abdominal mass. On evaluation with contrast-enhanced computed tomography abdomen and pelvis and image-guided biopsy he was diagnosed with mixed germ cell tumour of the right testis (predominantly a seminoma) with a retroperitoneal nodal mass and absent left testis, for which he received chemotherapy. Post-chemotherapy he underwent surgery and was diagnosed intraoperatively with PMDS along with TTE and testicular malignancy arising from the ectopic left testis. Postoperative recovery and follow-up were uneventful. Most cases of PMDS are diagnosed early in life. They present clinically with unilateral or bilateral undescended testis with inguinal hernia. In adults, PMDS is usually associated with male infertility. However, TTE is associated with an increased risk of testicular tumours if undiagnosed until adulthood. In adults PMDS with TTE is usually an intraoperative finding and is commonly associated with malignancy in the ectopic/undescended testis.

## Background

Persistent Müllerian duct syndrome (PMDS) is a rare sexual development disorder and is even more rarely associated with transverse testicular ectopia (TTE). In PMDS a normal male genotype (46XY) has a uterus and fallopian tubes. PMDS is caused by the lack of anti-Müllerian hormone (AMH) or AMH-2 receptor deficiency. TTE is a rare form of testicular ectopia, in which both testes descend through a single inguinal canal and are present in the same hemiscrotum.^1^ TTE is associated with an 18% risk of malignancy in the gonads.^2^ Here we report the case of a 48-year-old man who presented to our institute, a mission-based multispecialty academic hospital, and was diagnosed with a large irreducible right inguinal hernia with testicular malignancy, and who was found to have PMDS and TTE at the time of the surgery. This case has been reported in line with the SCARE criteria.^3^

## Case history

**Figure 1:**
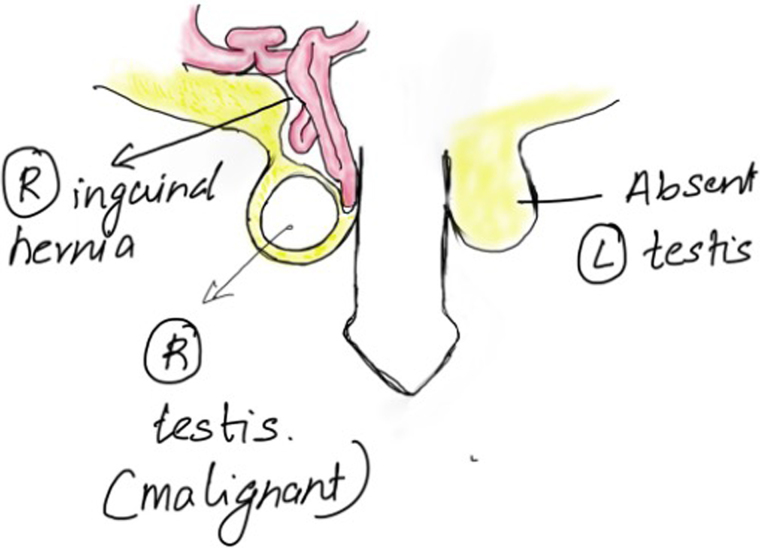
Diagrammatic representation of clinical presentation

A 48-year-old man presented with complaints of a large right inguinoscrotal swelling, backache and a significant weight loss (10kg) over six months. The patient had diabetes, was hypertensive and was a chronic smoker with chronic obstructive pulmonary disease. On clinical examination, he was found to have a complete, irreducible right inguinal hernia and an enlarged, hard right testis with an absent left testis. He was also found to have a hard, fixed, central abdominal mass (Figure 1).

**Figure 2:**
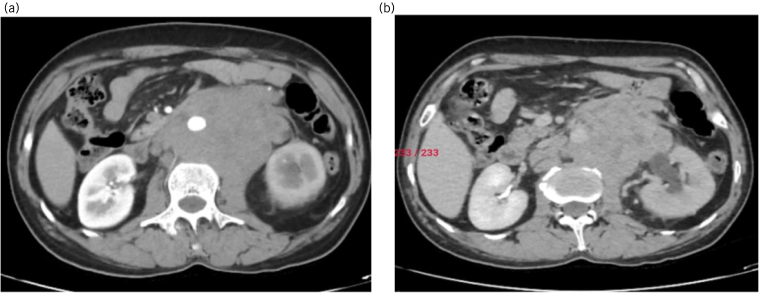
Contrast-enhanced computed tomography abdomen and pelvis scan showing a retroperitoneal lymph nodal mass (a) encasing the aorta and (b) the effect of pressure on the left ureter resulting in hydroureteronephrosis.

In view of these clinical findings, the patient was suspected of having testicular tumour of the right testis with retroperitoneal lymph nodal mass and was evaluated further for the same with tumour markers and imaging studies. Serum lactate dehydrogenase was elevated to 496iu/l. However, beta-human chorionic gonadotrophin (24μiu/ml) and alpha-fetoprotein (2.54iu/ml) were normal. Contrast-enhanced computed tomography (CECT) of the abdomen, pelvis and chest was done, which showed a neoplastic lesion of the right testis with a large lobulated nodal mass measuring 8.1 × 10.2 × 11.4cm extending from L1 to L3, encasing the aorta, inferior mesenteric artery, left renal artery and renal vein, abutting and compressing the inferior vena cava with pressure effect on the proximal part of the left ureter causing hydroureteronephrosis, absent left testis and right inguinal hernia with small bowel as content. Following the above investigations, the patient was diagnosed with right testicular malignancy with lymph nodal metastasis, right inguinal hernia and an absent left testis (Figure 2).

**Figure 3:**
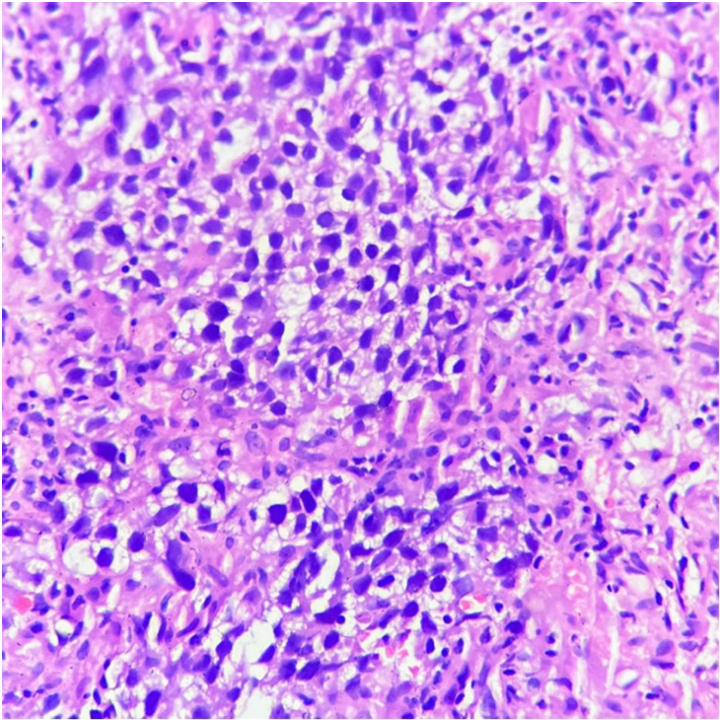
Germ cell tumour: seminoma (haematoxylin and eosin, ×400)

A multidisciplinary tumour board (MDT) discussion was held and a treatment plan of right high inguinal orchidectomy with inguinal hernia mesh repair was made. During preanaesthesia work-up and evaluation, the patient was found to have hypercalcaemia with electrocardiogram changes. Because of this, clearance to go ahead with the surgery was not given. After repeat discussions in the MDT regarding the same, a change in plan was made and the patient underwent an ultrasound-guided biopsy of the retroperitoneal nodal mass under local anaesthesia, so that chemotherapy could be started at the earliest opportunity. The biopsy was reported as predominantly seminoma with a focal area of embryonal carcinoma – mixed germ cell tumour (Figure 3). The final pretreatment diagnosis was right testicular mixed germ cell tumour with retroperitoneal lymph nodal metastasis, absent left testis and a complete right inguinal hernia with small bowel as content.

**Figure 4:**
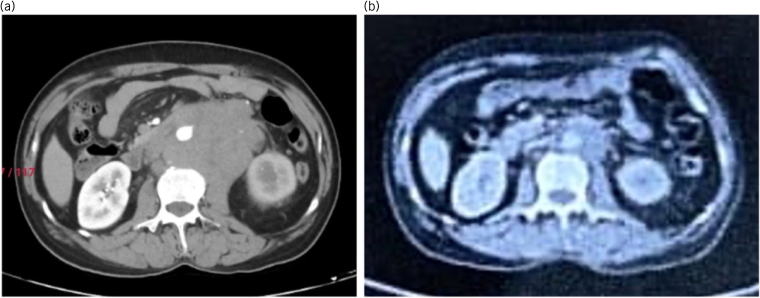
(a) Pre-chemotherapy contrast-enhanced computed tomography abdomen and pelvis scan showing a large retroperitoneal lymph nodal mass. (b) Post-chemotherapy positron emission tomography–computed tomography scan showing a significant reduction in the size of the retroperitoneal lymph nodal mass.

The patient was started on a bleomycin, etoposide, cisplatin (BEP) regimen and received three cycles of BEP. During chemotherapy, there was deterioration of his lung function, confirmed with a pulmonary function test. Therefore, the regimen was changed and instead of a fourth cycle of BEP, the patient received cisplatin, etoposide and ifosfamide. Post-chemotherapy the patient was reassessed with a positron emission tomography–computed tomography (PET–CT) scan, to determine the response to chemotherapy. The PET–CT scan showed a residual 3.5 × 3.2 × 5.2cm retroperitoneal mass with mild ^18^F-fluorodeoxyglucose (FDG) uptake, and residual mild FDG uptake in the right hemiscrotum, indicating a good response to chemotherapy (Figure 4). In view of a residual metabolically active retroperitoneal nodal mass of >3cm, the patient was planned for surgery – a right high inguinal orchidectomy and retroperitoneal lymph node dissection, along with right inguinal hernia meshplasty. After adequate preanaesthesia work-up and preoperative optimisation, he was taken up for the surgery.

**Figure 5:**
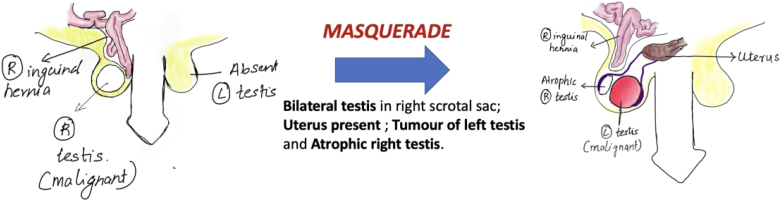
Diagrammatic representation of the masquerade

**Figure 6:**
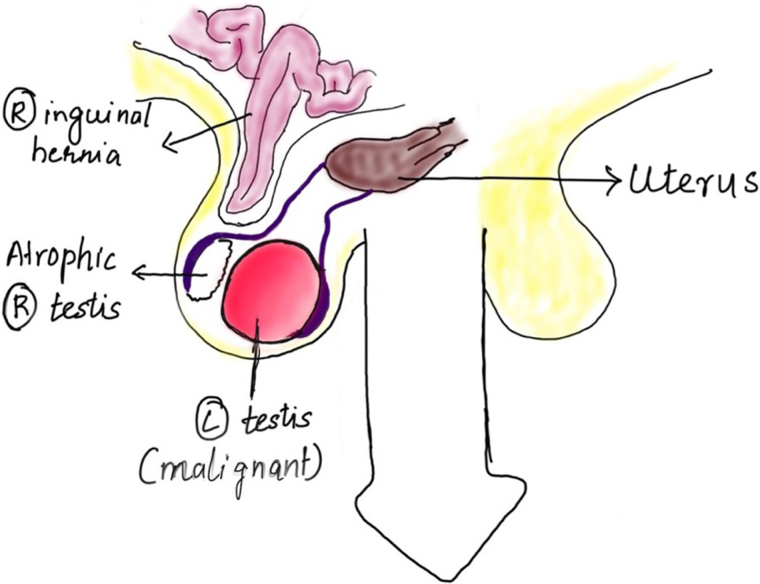
Diagrammatic representation of intraoperative findings

**Figure 7:**
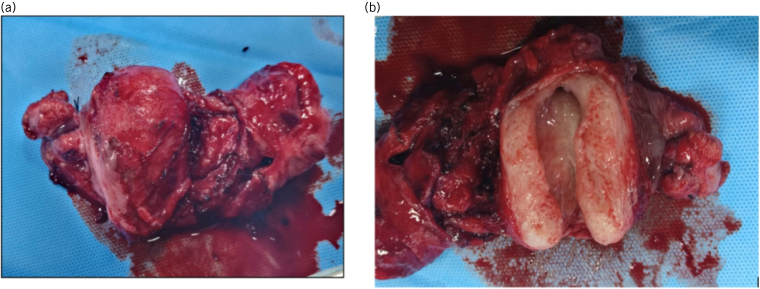
(a) Müllerian structures: uterus with fallopian tubes. (b) Cut section showing endometrial cavity.

**Figure 8:**
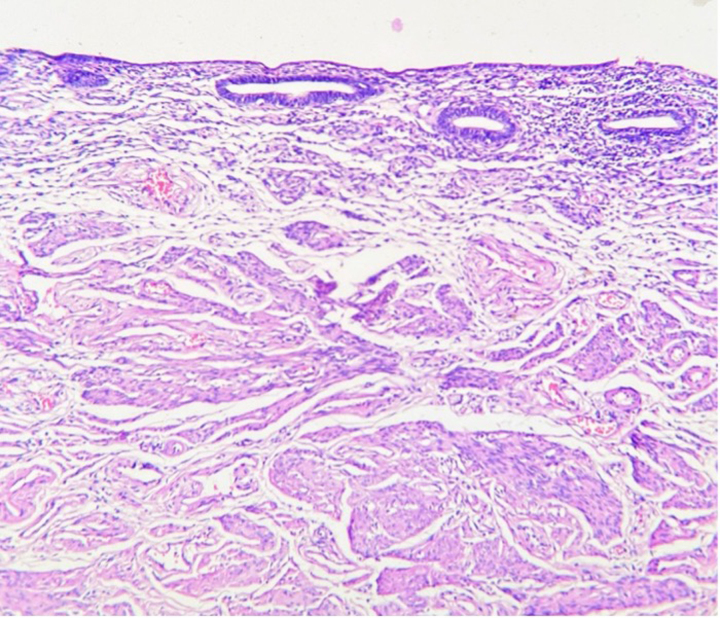
Uterine structure showing endomyometrium (haematoxylin and eosin, ×100)

**Figure 9:**
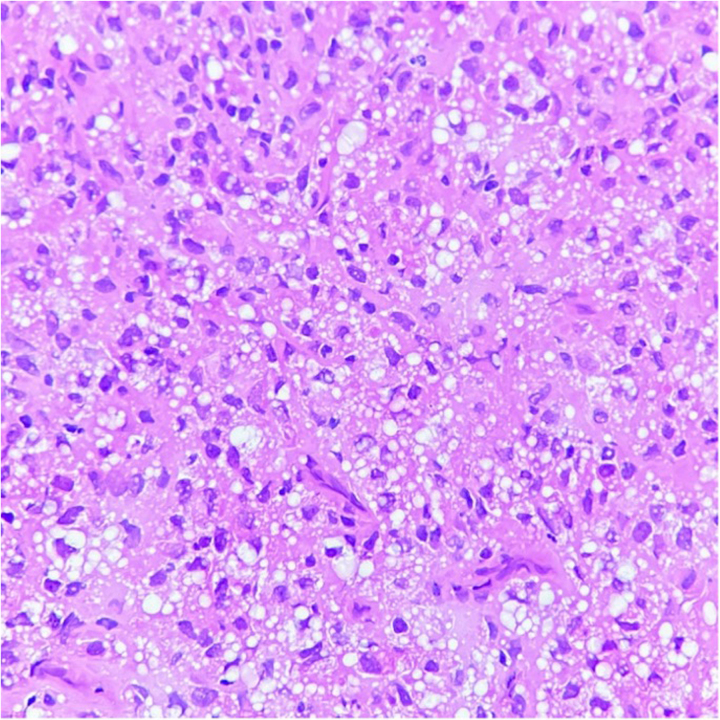
Post-chemotherapy image showing no viable tumour. Inflammatory cells and histiocytes are present (haematoxylin and eosin, ×400).

The entire ‘masquerade’ was revealed during the surgery (Figure 5). Intraoperatively, both testes were found to be in the right hemiscrotum. The tumour was actually found to arise from the left testis and the right testis was atrophic. The large hernia sac was adherent to the right testis. On tracing the testes proximally, they were found to be attached to a uterus-like structure. Therefore, a diagnosis of PMDS with TTE was established (Figure 6). The patient underwent left orchidectomy and retroperitoneal nodal dissection for the testicular malignancy along with removal of the Müllerian structures and right inguinal mesh hernioplasty. The cut section of the Müllerian structure confirmed it to be a uterus with an endometrial cavity and fallopian tubes attached to it (Figure 7). The final histopathology reported no residual viable tumour in the left testis or the retroperitoneal nodal mass along with the histopathological confirmation of a uterus (Figures 8 and 9). The patient had an uneventful postoperative period and recovered well. He is on regular follow-up and free of disease at the end of six months.

## Discussion

PMDS is characterised by the persistence of the Müllerian duct structures in a karyotypical male.^4^ Based on the location of testes, PMDS can be classified into three groups. The first involves bilateral intraabdominal testes in a position analogous to ovaries, which is seen in 60%–70% of cases of PMDS. In the second group, one testis is seen in a hernial sac or scrotum with a contralateral inguinal hernia; this accounts for 20%–30% of cases. Finally, in the third group, which accounts for 10% of cases, both testes are seen in the same side, along with fallopian tubes and uterus.^5^ TTE is also divided clinically into three types: type 1, the most common, accounts for 40%–50% of cases and is associated with inguinal hernia; type 2 is associated with PMDS (30%); and type 3 is associated with other anomalies, for example scrotal, hypospadias or renal (20%).^6^ PMDS in combination with TTE is rare. Very limited cases, fewer than 300, of PMDS with TTE have been reported in the literature. The mechanism of TTE in PMDS is not clear. It could be due to the persistent Müllerian duct structures causing a mechanical effect, preventing normal descent of the testis and also leading to transverse ectopia across the midline. This rare entity was found in our case.

Most cases are diagnosed early in life. They present clinically with unilateral or bilateral undescended testis with inguinal hernia. In adults, it is usually associated with male infertility. However, TTE is associated with an increased risk of testicular tumours, if undiagnosed until adulthood. The overall risk of malignant transformation is around 18%–33%, with seminoma being the most common although others like embryonal carcinoma, yolk sac tumour and teratoma can also be found.^2^

TTE can be diagnosed before surgery with a thorough history and clinical examination, along with imaging like ultrasonography and CT abdomen. PMDS is usually diagnosed intraoperatively with the identification of a uterus and fallopian tubes. It also requires investigations such as karyotyping and sex hormones levels for confirmation.

The principle of treatment is to preserve fertility and place the testis in the hemiscrotum at the earliest, and to prevent malignant transformation. Excision of the Müllerian structures is not routinely required, because they do not have any risk of malignant transformation. However, they may need to be excised if they hinder orchidopexy or cause abdominal discomfort.^7^

In our patient, a preoperative diagnosis of testicular tumour from the descended right testis was made because the CECT did not show the presence of an ectopic or undescended left testis. However, intraoperatively the patient was found to have a combination of PMDS with TTE and testicular malignancy in the ectopic testis. The presence of these rare conditions was probably masked in our imaging studies by the large inguinal hernia, making it difficult for a preoperative diagnosis of these conditions. With the intraoperative findings, the patient underwent orchidectomy of the pathological ectopic left testis with orchidopexy of the normally descended right testis and excision of the Müllerian structures with an inguinal hernia mesh repair. Orchidopexy was done to preserve any residual testicular function in the normally descended right testis, also making it accessible for easy clinical evaluation on follow-up.

## Conclusions

This was an ‘unusual case’, encompassing PMDS with TTE along with malignancy arising from the ectopic testis. PMDS in combination with TTE is very rare. It is usually an intraoperative diagnosis and requires a very high suspicion for preoperative diagnosis.
